# Dense CO_2_ as a Solute, Co-Solute or Co-Solvent in Particle Formation Processes: A Review

**DOI:** 10.3390/ma4112017

**Published:** 2011-11-16

**Authors:** Ana V. M. Nunes, Catarina M. M. Duarte

**Affiliations:** 1 Requimte/CQFB, Departamento de Química, Faculdade de Ciências e Tecnologia, Universidade Nova de Lisboa, Campus de Caparica, Caparica 2829-516, Portugal; E-Mail: ana.nunes@dq.fct.unl.pt; 2 Instituto de Biologia Experimental e Tecnológica (IBET), Apartado 12, Oeiras 2781-901, Portugal; 3 Instituto de Tecnologia Química e Biológica, Universidade Nova de Lisboa, Avenida da Republica, Oeiras 2780-157, Portugal

**Keywords:** particle formation, supercritical CO_2_, particles from gas saturated solution

## Abstract

The application of dense gases in particle formation processes has attracted great attention due to documented advantages over conventional technologies. In particular, the use of dense CO_2_ in the process has been subject of many works and explored in a variety of different techniques. This article presents a review of the current available techniques in use in particle formation processes, focusing exclusively on those employing dense CO_2_ as a solute, co-solute or co-solvent during the process, such as PGSS (Particles from gas-saturated solutions^®^), CPF (Concentrated Powder Form^®^), CPCSP (Continuous Powder Coating Spraying Process), CAN-BD (Carbon dioxide Assisted Nebulization with a Bubble Dryer^®^), SEA (Supercritical Enhanced Atomization), SAA (Supercritical Fluid-Assisted Atomization), PGSS-Drying and DELOS (Depressurization of an Expanded Liquid Organic Solution). Special emphasis is given to modifications introduced in the different techniques, as well as the limitations that have been overcome.

## 1. Dense CO_2_ in Particle Formation Processes

Particle formation processes using dense gases have emerged within the last two decades as a promising alternative technology to overcome some technical problems and limitations related to the use of conventional methodologies [1,2,3,4,5,6,7]. The most used classical processes such as jet and ball milling, spray-drying, and recrystallization using solvent evaporation or liquid anti-solvent, do comprise several drawbacks like the presence of high shear forces, high temperatures, electrostatic charges and also the contamination of the final product with undesirable organic solvents [8,9]. Dense gas techniques can overcome most of these disadvantages by exploring the “unique” properties of fluids in the vicinity of the critical point. These properties include liquid-like densities, gas-like transport properties and an unusual high compressibility which allows adjustment of the solvent power of the fluid with minor changes in pressure and temperature [8,9,10,11]. 

Particularly in the case of dense CO_2_ (the most widely used dense gas) processes can be carried out at mild temperatures, due to CO_2_ low critical temperature, avoiding thermal degradation of labile compounds. Furthermore, the benign properties of CO_2_ (non-flammability and relatively low toxicity) and its ready separation from the products make CO_2_ the elected solvent for processing products for human consumption, which has generated a special interest from the pharmaceutical and food sectors, the main top target industries of this particle formation technology [10]. 

Depending on the technique, dense CO_2_ can totally or partially replace the use of harmful organic solvents, which is often highlighted as an important strategy within green chemistry and to enable new, clean technologies [12]. Several green chemistry principles are in fact satisfied, namely in what concerns pollution prevention, lower toxicity and the use of an abundantly available resource [13]. However, as it was pointed out by Beckman in 2004 [10], it is essential to assure that the use of CO_2_ can originate a product with superior characteristics providing a performance rather than just an environmental advantage, making of this technology an effective alternative to well established industrial processes. In this context, numerous scientific works have been published and the suitability of dense CO_2_ has been demonstrated both for the precipitation of pure compounds and composites, showing improved performances mainly in terms of reduction of particle size and distribution, as well as in terms of morphology control [14,15,16,17,18,19,20]. 

CO_2_ precipitation processes can be divided in two major groups, the first including operations that are driven by the solvent strength of CO_2_, where CO_2_ can act as a solvent as in Rapid Expansion from Saturated Solutions (RESS) or as an anti-solvent as in the Supercritical Anti-Solvent process (SAS). Briefly, in the RESS process, the solid substance to be micronized is dissolved in compressed CO_2_ and then rapidly depressurized through a nozzle with consequent precipitation of the substance due to the large experimented decrease of CO_2_ solvent power. In the SAS process, the substance of interest is dissolved in a classical solvent and precipitates when contacted with dense CO_2_ as a result of the supersaturation attained due to the large solubility of CO_2_ in most organic solvents. For the SAS process, different methodologies based on different mixing models between solution and SCF were subsequently developed as Gaseous Anti-solvent (GAS), Aerosol Solvent Extraction (ASES), and Solution Enhanced Dispersion by Supercritical Fluids (SEDS) [14].

The second group comprises all the operations that do not depend on CO_2_ solvent power but instead take advantage of the great volume expansion and the large cooling effect produced when CO_2_ is depressurized from operating conditions to ambient pressure as in Particles from Gas Saturated Solutions (PGSS) and subsequent developed processes, CPF (Concentrated Powder Form), CAN-BD (Carbon dioxide Assisted Nebulization with a Bubble Dryer^®^), SEA (Supercritical Enhanced Atomization), SAA (Supercritical Fluid-Assisted Atomization), PGSS drying and DELOS (Depressurization of an Expanded Liquid Organic Solution). 

This review will be focused exclusively in this second group in which CO_2_ can be used as solute, co-solute or co-solvent.

## 2. The Particles from Gas Saturated Solution (PGSS) Technique

**Figure 1:**
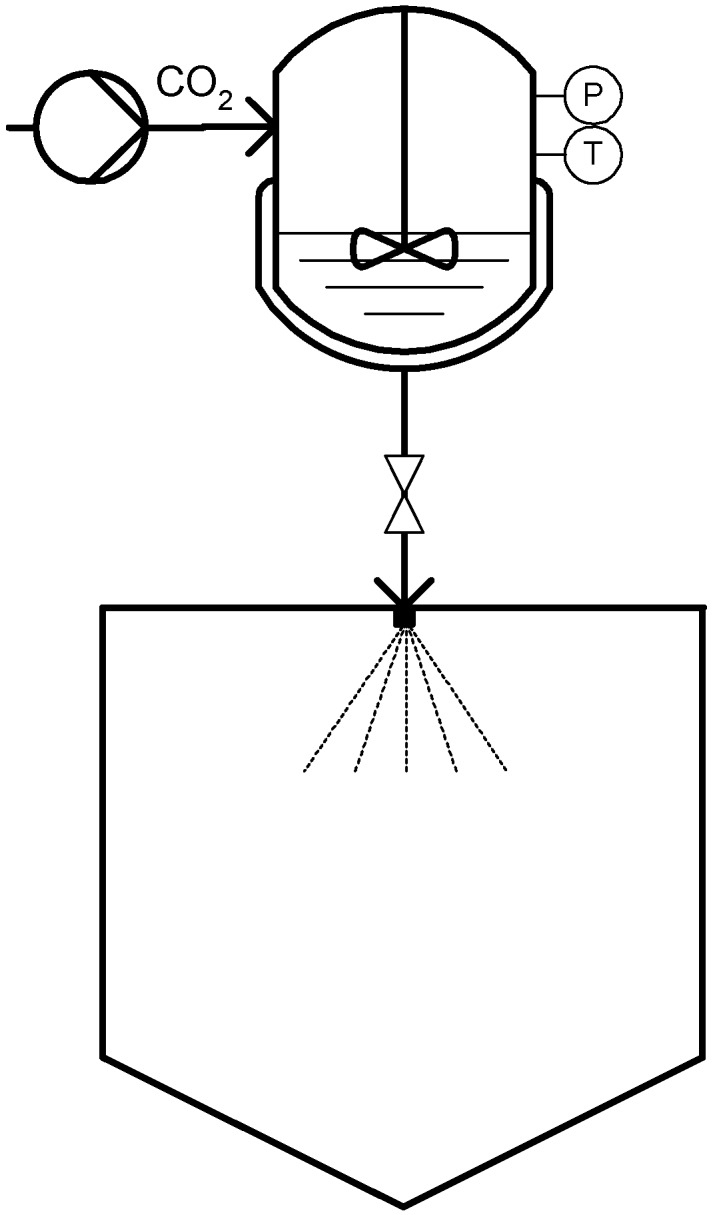
Particles from Gas Saturated Solution (PGSS) technique.

The PGSS technique was patented [21] by Weidner and co-workers in 1994 and presented [22] in the Third International Symposium on Supercritical Fluids in Strasbourg in the same year. It is considered one of the most attractive CO_2_ based micronization processes because it does not rely on the solvent strength of CO_2_, it employs relatively low operating pressures and can totally eliminate the need for organic solvents [9]. A schematic diagram of a typical PGSS process is presented in Figure 1. The process consists in dissolving the compressed gas into the molten material in a stirred high pressure reactor until saturation is reached. The gas-saturated solution formed which can typically contain between 5–50 wt % of the compressed gas is then expanded through a nozzle and solid particles are formed due to the extremely rapidly temperature decrease caused by the fluid expansion that is commonly known as the Joule-Thomson effect [20].

**Figure 2:**
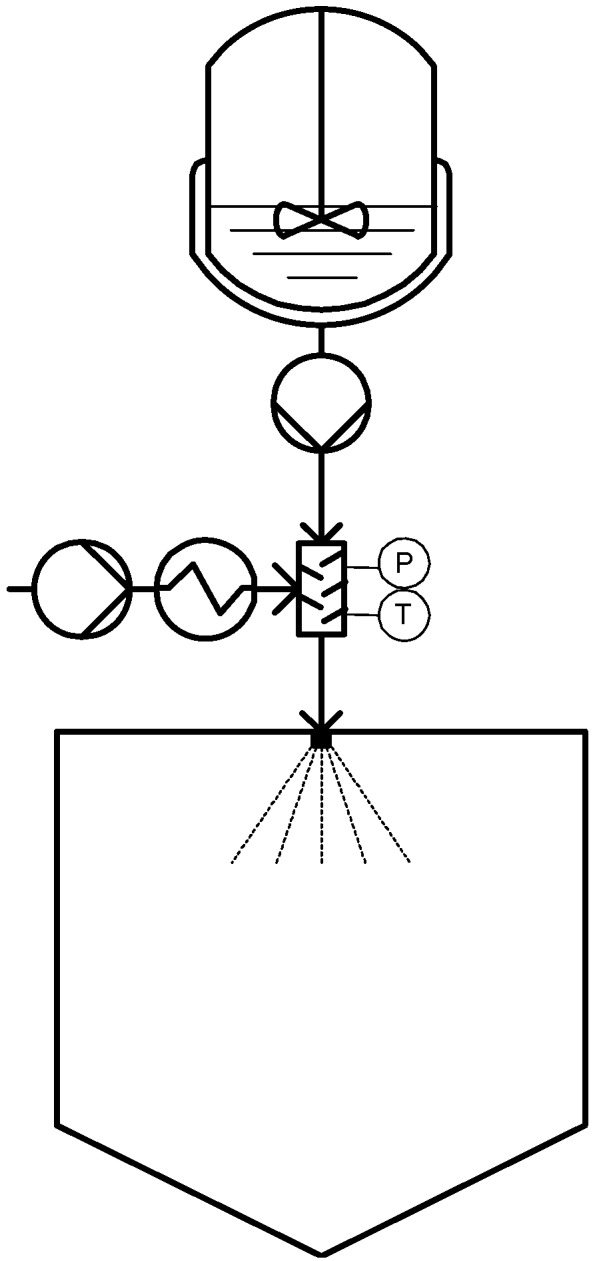
Continuous PGSS technique.

The PGSS process can also be operated in a continuous mode in which the solute of interest is fed in the molten state via a pump and at the same time pressurized CO_2_ is introduced into that pipe. Intensive mixing between the two streams is achieved in a static mixer. After the mixing zone, the mixture is expanded through a nozzle [20,23]. A schematic diagram of a PGSS process in continuous mode is presented in Figure 2. 

This process is especially suitable for processing polymers and lipids in which CO_2_ has a large solubility. Moreover, since it has a melting point depression effect, substances can be sprayed, which, under classical conditions can hardly be sprayed or even not be sprayed at all [20]. The extent of melting point depression experimented by each substance depends on the amount of CO_2_ that solubilizes into the substance and is caused by molecular interactions between dissolved CO_2_ and the substance of interest [6]. Determination of solid-liquid transitions in pressurized systems is essentially as it gives information on the pressure needed to melt the substance to be micronized and form a liquid phase at a given temperature [24,25]. 

The first PGSS reported application was for the generation of powders from Polyethylene glycols (PEGs) [26]. PEG is a widely used hydrophilic polymer due to its biocompatibility and non-toxicity; it is used as a carrier material in the development of pharmaceutical and cosmetic formulations and was used by Weidner and co-workers to improve understanding on how process parameters influence final product properties. For this purpose, dependencies of particle size distribution, morphology and bulk density on process parameters like pre-expansion pressure, pre-expansion temperature and gas to product ratio (GTP) were studied [20]. The authors found out that smaller particles are formed with increasing pressure and GTP ratios and that, for higher GTP ratios, the pressure influence is less pronounced. Particle morphologies are strongly influenced by pre-expansion temperature and can actually be tailor made in a range between 3 to 500 µm with bulk densities from about 90 kg/m^3^ up to 600 kg/m^3^, by applying different operating conditions [19,27]. The technique concept has already proven it feasibility even at the economical level (often considered as the major obstacle to SCF industrial application) and reached the industrial scale, which is a big advantage over other technologies that are still under development [20]. Nevertheless, some fundamental issues still require further research in order to build theoretical models, for example, for the mixing process under pressure, the spray generation in the nozzle and the solidification kinetics of the substance [20].

Main limitations of the PGSS process is that the solute has to be melted, which can be problematic for heat sensitive materials [1,8]. In order to overcome this limitation, the technique has also been applied to process suspensions of active substances in low melting polymers or other carriers to produce composite particles mainly containing bioactive compounds [18,28,29,30] and also for coating applications [31,32,33,34,35,36]. Different strategies can be used to improve the process performance depending on the difficulties experimented and that are in most of the cases inherent to the systems under investigation. Hao *et al.* [37] reported the use of a nitrogen back pressure to suppress the loss of CO_2_ from the P_DL_LA/CO_2_ liquefied mixture and in that way slow down the rate of polymer solidification to achieve the production of fine particles. The authors have also cooled the collection chamber with liquid nitrogen to prevent aggregation of the newly formed microparticles. In addition, Salmasso *et al.* [38,39] described a variation of the PGSS technique that was reported by the authors as the Gas Assisted Melting Atomization (GAMA) process, in which the introduction of a co-axial air injection device in the typical PGSS precipitation vessel facilitated the yield of insulin-loaded solid lipid submicron particles and avoided agglomeration.

## 3. Role of Dense CO_2_ in PGSS Related Techniques

### 3.1. Solute: CPF and CPCSP

After Weidner patented the PGSS technique, several modifications were introduced in the process by the former and by other authors in order to extend its applications and/or to overcome the main process limitations. 

**Figure 3:**
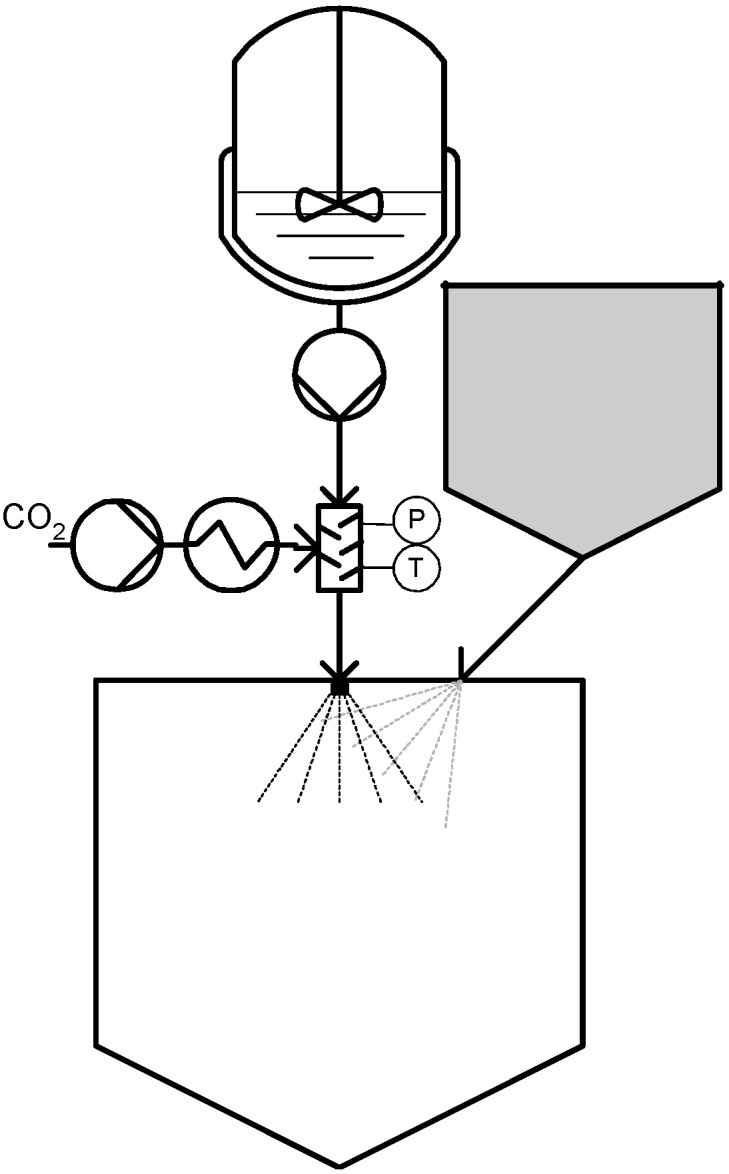
Concentrated Powder Form (CPF^®^).

In 1997, CPF^®^ (Concentrated Powder Form) was proposed by Weidner [40,41] which allows for the generation of powders containing an unusually high content of liquids. In the CPF process, a liquid substance is contacted with the dense gas and depressurized through a nozzle. Instead of solid particles, a dispersed spray of fine droplets is formed during the expansion step of the gas-saturated liquid. A powdered carrier material is then blown into that spray by means of an inert gas, which binds the droplets, so that a free flowing powder that can contain 90 wt % (or more) of liquid is formed [20]. Figure 3 represents a schematic diagram of a CPF process.

It should be noted that, unlike original patented PGSS, precipitation does not occur at any point of the process, instead, particles are formed either by infiltration of droplets into porous materials or by agglomeration of non-porous material, depending on the carrier used. This process has been applied to more than 100 liquids and 60 different solid carriers and is already being performed at an industrial scale with a capacity of 300 kg/h of powder production. According to Weidner, powders produced on this scale are mainly liquid extracts of essential oils which are converted by the CPF^®^ process into an easily dosable powder with standardized quality and a long shelf life [20].

**Figure 4:**
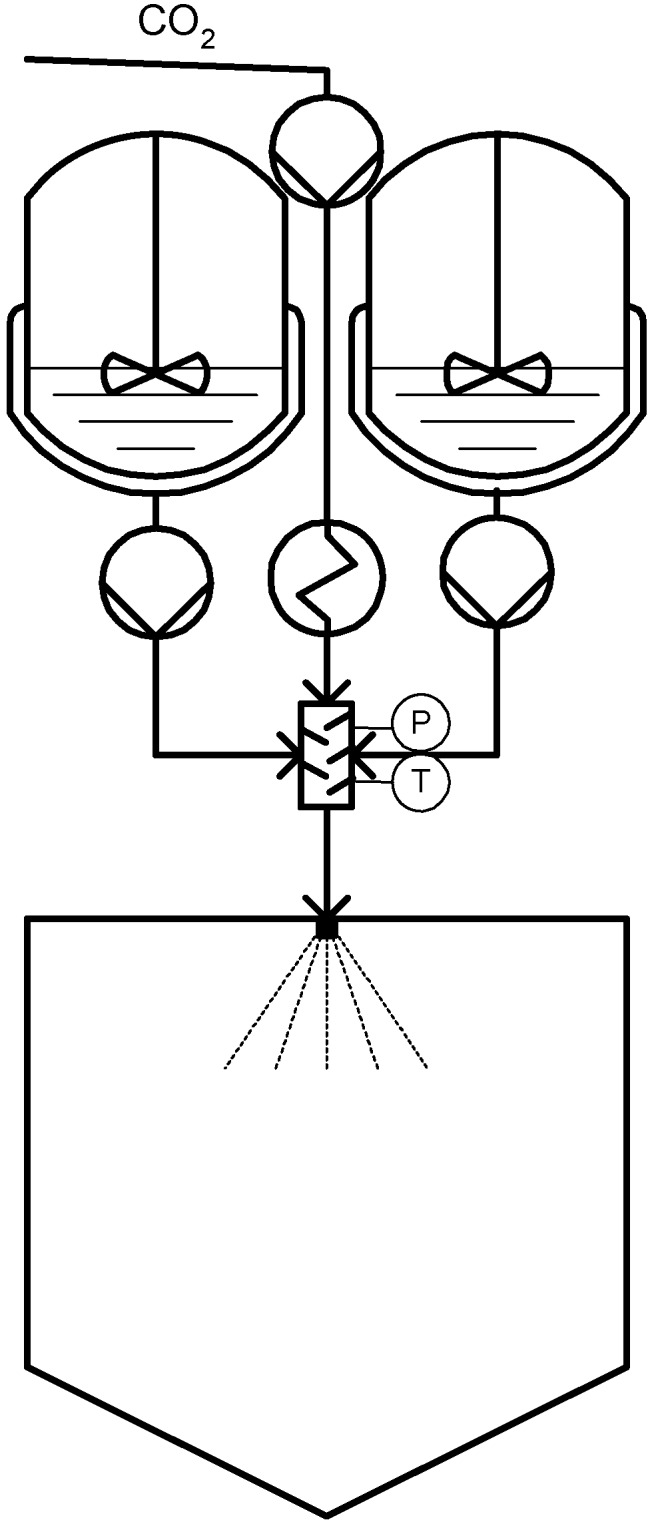
Continuous Powder Coating Spraying Process (CPCSP).

Another PGSS-derived process was proposed by Weidner in 1999 as an alternative technique for the manufacture of powder coatings [42]. The conventional technique involves high temperature stress of the coating system composed by two polymers, the binder and the hardener, as well as long residences times which may cause a premature reaction that would destroy the product [43]. In the Continuous Powder Coating Spraying Process (CPCSP), the binder and hardener are melted in separate vessels to avoid premature reaction of the polymers as illustrated in Figure 4. Both melted components are then admitted to a static mixer and are homogenized with compressed CO_2_. Due to dissolved carbon dioxide, the melting point of the mixture decreases and thus, it is possible to perform homogenization at very low temperatures and using very short residence times and in this manner avoid reaction. The solution formed in the mixer is expanded afterwards via a nozzle into a spray tower. Due to CO_2_ volume expansion and consequent drastic temperature decrease, a fine powder coating is formed. With a blower, the gas is removed from the spray tower and by means of a cyclone and a filter the fine particles are separated from the gas [43].

Weidner *et al.* [43] have effectively applied this technology to low melting polyester powder coatings with an average particle size of less than 40 μm, which is not possible to achieve by manufacturing the coating powders using a conventional process.

### 3.2. Co-Solute (and Propellant): CAN-BD^®^, SAA, SEA and PGSS-Drying

Sievers *et al.* [44,45] proposed a modification to the PGSS technique which allowed expanding the process application to the use of any compound that is water soluble, greatly increasing the range of substances that can be processed. This patented process, known as Carbon Dioxide Assisted Nebulization with a Bubble Dryer (CAN-BD)^®^, involves the mixing of a liquid stream (generally aqueous although it can also be organic) containing the drug and any excipients or stabilizers (typically 1 to 10% of total solids dissolved) and a stream of a compressed CO_2_ to generate a gas-liquid emulsion or solution. This solution is then decompressed through a flow restrictor forming a primarily aerosol of microbubbles and microdroplets which are further break by the expansion of dissolved CO_2_ in the liquid. Because CO_2_ is one of the most soluble gases in water (1.6 mole % at 63 °C and 100 bar), its use enhances the expansion process [46,47,48]. This aerosol is introduced into a classical spray tower to be dried by means of a conventional drying gas (air or nitrogen). 

This process generates particles less than 5 μm in diameter by rapidly drying of aerosolized solutions at relatively low temperatures (typically between 5 °C and 65 °C) [48]. It is claimed that the use of CO_2_ facilitates the formation of extremely fine droplets that dry faster than aerosol formed with conventional methods. In this way the temperature required to dry the nebulized solutions are lower than those required in the spray-drying technique thus making this technique more suitable for processing thermally labile compounds [9]. 

**Figure 5:**
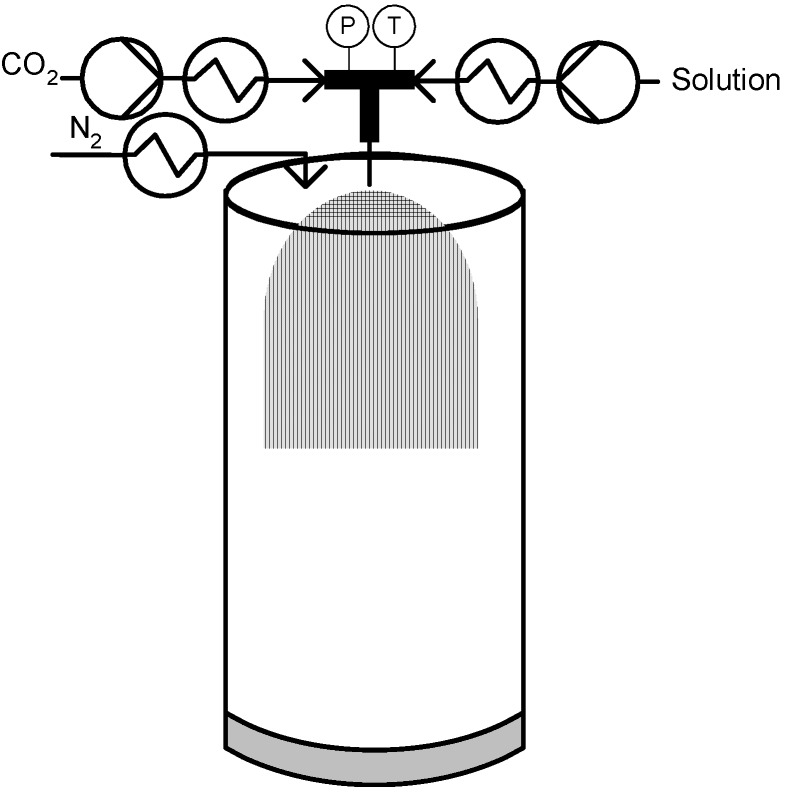
Carbon Dioxide Assisted Nebulization with a Bubble Dryer (CAN-BD)^®^.

Different variants for contacting the liquid solution with CO_2_ are described by the inventors. The most cited variation, schematically presented in Figure 5, is the dynamic approach in which the biphasic mixture of the compressed gas and the aqueous solution is generated in a low-dead-volume (<1 µL) mixing tee. Alternatively, a static approach is described in which the compressed gas is introduced in a vessel containing an aqueous solution and mixed. The mixture is then decompressed through the restrictor [14,47]. 

This technique seems to have a lot of advantages since it is very simple and can be applied to a large range of substances, namely proteins and pharmaceuticals. Applications of this technique were extensively revised by Perrut in 2001 [14], by Cor Peters in 2003 [15] and by Sievers in 2008 [48]. Since original patent in 1997, Sievers and co-workers have proposed other applications and also some modifications in order to make CAN-BD process more versatile. The first extended application was the use of this technique in the production of powders of drug alcoholic solutions [49,50]. An additional modification was later proposed which uses a low-volume mixing cross instead of a tee that allowed mixing two liquid solutions (a water-based and an organic solvent-based solution) with scCO_2_ in order to generate composite particles [51]. More recently this technique has been applied by Sievers to the micronization of vaccines [52,53].

In 2002, Reverchon and co-workers introduced some modifications to the process developed by Sievers, particularly to the dynamic process, which allow improving the efficiency of mixing between CO_2_ and the liquid solution [54]. As mentioned by several authors, in the dynamic variant of the CAN-BD process, it is not clear to which extent fluid dissolution occurs and if saturation is in fact achieved due to the extremely short contact time in the tee [5,14,55]. Sievers showed by the acidity of the droplets formed, that at least some dissolution occurs and stated that the dispersion is not at all as efficient when CO_2_ is substituted by nitrogen or air at similar pressure [14].

In this context, Reverchon [56] proposed a different process setup to the one described by Sievers. The process intends also to deal with compounds that are water soluble but with several modifications. Main alteration proposed is the use of a saturator instead of a micrometric volume tee to obtain the mixing between the aqueous stream and the dense gas. The saturator loaded with stainless steel perforated saddles was design to provide a contacting surface and a residence time sufficient to allow the dissolution of SC-CO_2_ in the liquid solution up to the saturation conditions at the pressure and temperature of processing [54].

**Figure 6:**
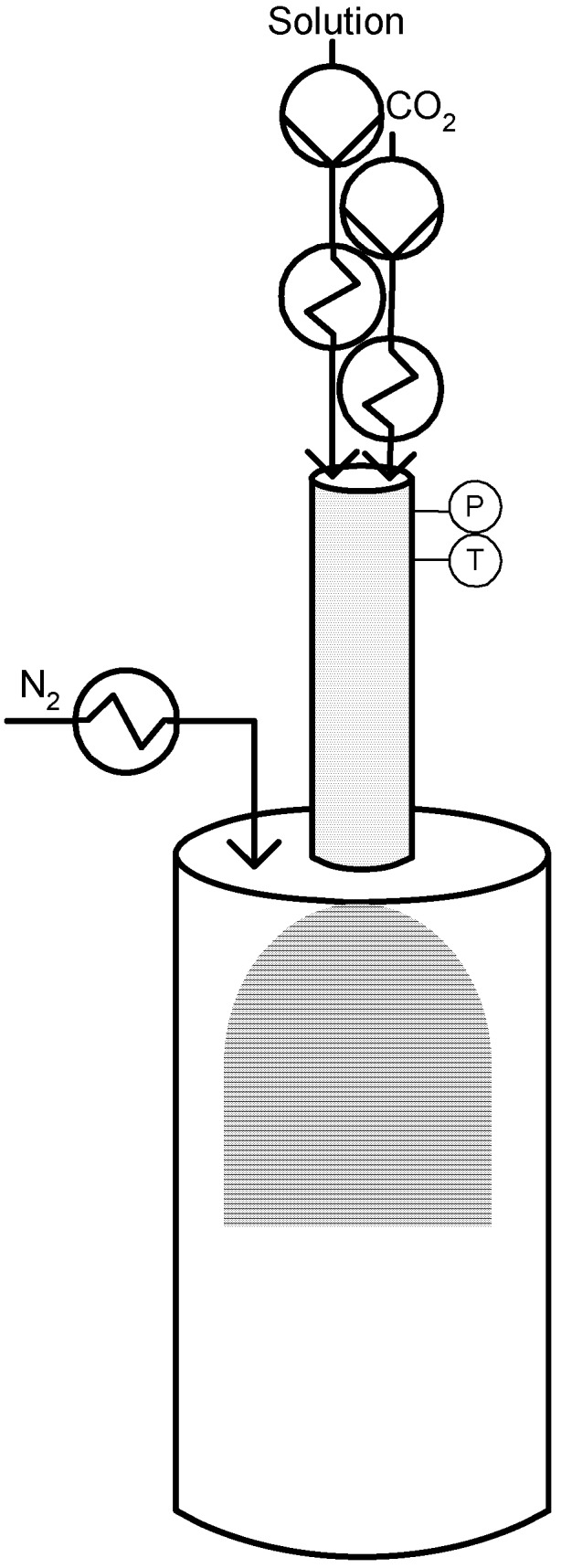
Supercritical Assisted Atomization (SAA).

The other modification introduced was the elimination of the capillary tube to avoid the problem of pressure drop and possible capillary blockage; instead, the liquid solution formed on the contraction device is sent to a thin wall injector. The injector produces a spray that forms the droplets in the precipitator, where a flow of heated N_2_ is introduced to facilitate the evaporation of the liquid solvent [54]. Figure 6 illustrates a schematic diagram of the SAA process.

Reverchon mentions that experiments were immediately successful using not only water but also organic solvents and that the SAA technique provides a good control over particle size producing microparticles sizing between 0,5 and 5 µm. The process parameter that mainly controlled the particle size in the SAA process was the concentration of the liquid solution [54]. The main applications of the SAA process were published by Reverchon and co-workers to the micronization of superconductors, ceramics and catalysts precursors, cyclodextrins and several drug compounds and also antibiotics using different liquid solvents [9,14]. More recently, the process was adapted to produce polymer particles and also drug-polymer composite particles [57]. 

As in the CAN-BD process, in the SAA, atomization takes place in two steps. In a first step, primary droplets are produced at the outlet of the injector and in a second step this droplets are divided in secondary droplets by the CO_2_ expansion from the inside of primary droplets. These secondary droplets are rapidly dried by warm N_2_ and solid particles are formed on the basis of a one droplet-one particle mechanism [58]. The main differences between CAN-BD and SAA processes are the region where the mixing is achieved and the extent of solubilization of scCO_2_ in the liquid solution where the solute is dissolved [6]. In both processes, CO_2_ acts as a co-solute during the process although it role is actually to assist the spraying of the liquid solutions by atomizing the solution which may not succeed if an eventual anti-solvent effect occurs. On the other hand, if the liquid massively solubilizes in the fluid phase, the remaining liquid solution saturates and solid precipitation starts in the contactor and the process fails. Thus, vapor liquid equilibrium data of the binary (CO_2_ + liquid solvent) systems of interest is an essential information in order to properly select operating conditions (P and T) that can guarantee a limited solubility of CO_2_ in the liquid and a small solubility of liquid in CO_2_ [50]. In 2008, Cai *et al.* [59] introduced a hydrodynamic cavitation mixer (HCM) in the SAA process in order to improve mass transfer between CO_2_ and liquid solution. The SAA-HCM process was successfully used to micronized levofloxacin hydrochloride and the influences of several process parameters were investigated and reported by the authors.

Another variation of the CAN-BD process was explored by Rodrigues *et al.* [60] The Supercritical Enhanced Atomization (SEA) process is also based on the supercritical fluids ability to enhance liquid jet dispersion into fine droplets when depressurized simultaneously with liquid solutions [61,62]. The authors emphasize that, in contrast to SAA, this process setup is not intended to saturate the solution with the supercritical fluid, which is also not likely to happen in the CAN-BD process. The main difference between this and the CAN-BD process is the utilization of a co-axial nozzle with a pre-expansion mixing chamber instead of the micrometric volume tee. The pre-expansion mixing chamber allows the mixing of both fluids at selected conditions of pressure and temperature prior to its depressurization into a precipitation vessel at atmospheric pressure [60]. An interesting feature of this setup is that the precipitation mechanism can be switch from atomization and droplet drying to anti-solvent precipitation, just by properly selecting the operational conditions of the mixture in the mixing chamber. When conditions are selected in a way that CO_2_ acts as an anti-solvent, the process is similar to a SEDS process. This setup was used by the authors to investigate the different morphologies that can be obtained for the micronization of lysozyme by changing the governing mechanism for precipitation from spray drying (spherical particles) to anti-solvent (production of fibers was favored). The authors further report that lysozyme activity was severely affected when an anti-solvent effect was observed. Finally, due to the high gas/liquid ratios involved, the setup makes no use of a secondary gas flow inside the precipitator for solvent drying as used by both CAN-BD and SAA techniques [60].

In addition, in a different approach called PGSS-drying [63], particles do not need to be dried by means of a flow of heated N_2_ as in CAN-BD and SAA techniques. Instead, the solvent is removed from the spray tower together with the gas and a free flowing powder precipitates at low temperatures (30–60 °C), in an inert oxygen-free atmosphere, which is particularly important when processing sensitive substances. As in the original PGSS process the liquid solution to be dried is intensively mixed with scCO_2_ using a static mixer at the desired temperature and pressure, except that, at this stage, despite the dissolution of some CO_2_ in the liquid solution, also a considerable amount of the solvent is extracted into the gas. This biphasic mixture is then sprayed through a nozzle into the spray tower where fine droplets are formed (due to CO_2_ expansion because of atmospheric conditions) and, in addition, evaporation of the residual solvent takes place (due to pre-selected temperature conditions in the spray tower) [20]. In order to achieve an efficient evaporation of the solvent in the spray tower, temperature has to be carefully selected in a way that both solvent and gas form a homogeneous phase which will then be exhausted by a blower from the spray tower. To achieve this homogeneous gaseous phase, post-expansion temperature must therefore be at least higher than the dew point of the binary system gas and solvent [64,65]. If the liquid solvent used is a mixture then phase equilibrium data of the multi-component system is necessary for determining suitable conditions for successfully performing the precipitation [66]. At the end of the process, a free flowing powder is collected at the bottom of the spray tower. As for the original patented PGSS process, the authors used PEG as a model substance to analyze the fundamentals of the process, discuss mass and energy balances, phase equilibrium conditions, mass transfer rates and atomization mechanisms [67]. Furthermore, a detailed experimental analysis of the influence of different process and design parameters (temperature, pressure, flow rates, design of the static mixer used to put into contact aqueous solution and CO_2_) has been carried out [68].

Although very promising, there are not many published applications of this technique, which has been explored by the inventors, mainly for industrial purposes [20]. The PGSS-drying was patented by Weidner in 2000, but first scientific publication of this process was only later reported by the author and co-workers in 2008 for the drying of aqueous green tea extracts [66]. It is to be expected that, in the next few years, several other applications will emerge, since besides being very promising, this technique already exists at a pilot stage and on an industrial scale, providing a basis for the demonstration of its technical and economic feasibility for industrial applications [20]. Very recently Cocero and co-workers have applied the PGSS-drying technique to encapsulate an essential oil, lavandin, in n-octenyl succinic modified starches [69]. 

### 3.3. Co-Solvent: DELOS

**Figure 7:**
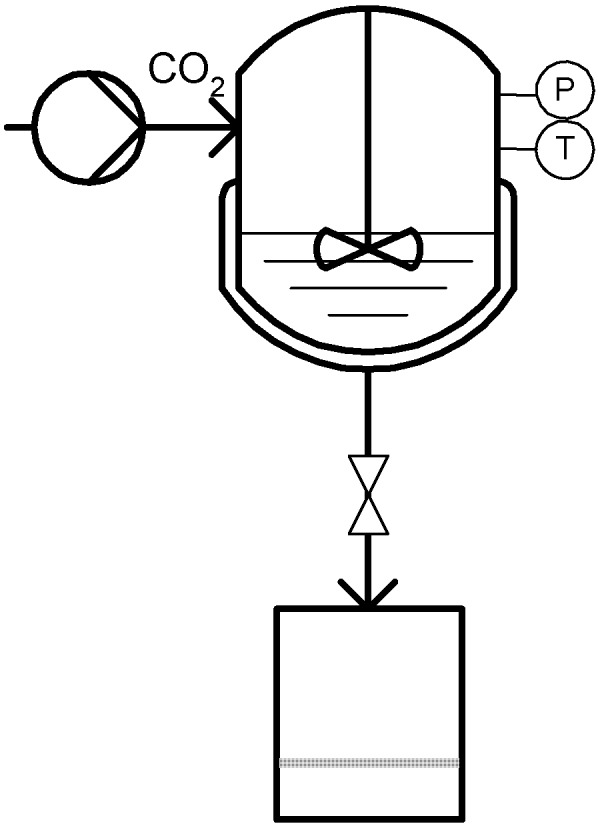
Depressurization of an Expanded Liquid Organic Solution (DELOS).

Ventosa *et al.* developed the DELOS (Depressurization of an Expanded Liquid Organic Solution) process [70]. In this case the compressed gas (e.g., CO_2_) is used to saturate an organic solution of the solute of interest, forming a volumetric gas expanded liquid solution. This solution is further expanded through a non-returning valve to atmospheric pressure experiencing a large temperature decrease due to pressure reduction and consequent CO_2_ expansion, which causes the precipitation of submicron or micron-sized particles with a narrow particle distribution [71]. A schematic diagram is shown in Figure 7.

The inventors describe the process in three sequence stages comprising initially the dissolution of the solute of interest in a conventional organic solvent at atmospheric pressure and operating temperature with a concentration bellow the saturation limit. The next step consists of pressurizing the organic solution by adding dense CO_2_ until working pressure is reached. An expanded liquid solution is formed with a certain molar fraction of dissolved gas, which the inventors term the working composition. The operative pressure should not exceed the critical point of the CO_2_/solvent mixture. Finally, the gas expanded solution is depressurized over a non-returning valve to atmospheric pressure, keeping the upstream pressure constant with N_2_. Particles are collected after cleaning of the precipitates with CO_2_.

It should be noted that, in this process, the compressed gas acts as a co-solvent being completely miscible at a given pressure and temperature with the organic solution of the solute to be crystallized [71]. An undesired anti-solvent effect of CO_2_ is therefore a possibility to be considered at stage 2, as if it occurs; the solute will precipitate in the saturator and not in the expansion vessel [15]. The solute concentration at this stage should therefore remain below the saturation limit in the expanded solution. The knowledge of the solute solubility behavior in CO_2_ expanded solvent is, however, of crucial importance to successfully employ the DELOS process [72]. This process can only be applied to substances for which the anti-solvent effect of CO_2_ is small [8].

Using the DELOS process, Ventosa *et al.* [71,72] have obtained particles less than 5 μm with a narrow size distribution and a high degree of cristalinity of a colorant powder, (1,4-bis-(n-butylamino)-9,1) anthraquinone. The authors found out that the size of the particles is a function of the magnitude of temperature decrease in the third stage [9], which is ruled by the working composition of the ternary mixture before depressurization [73]. The greater the decrease in temperature experienced by the solution, the smaller the particles obtained. For this specific substance, the authors also found that the DELOS process was more efficient than the GAS process [72]. In fact, smaller particles with a narrower size distribution were obtained which the authors explained to be due to the fact that the system solute/acetone/CO_2_ exist in one phase liquid solution for a wide range of CO_2_ molar fractions and added that, in systems with this behavior, the DELOS process is an alternative to the GAS process. The authors do not mention the existence of stirring in the high pressure vessel, actually they even refer the possibility of introducing CO_2_ through the bottom of the vessel in order to ensure faster CO_2_ dissolution rate and add that the design of the stirring system is not important because the characteristic of the particles produced do not depend on the mixing efficiency.

A modification to the DELOS process, called DELOS-SUSP [74] patented in 2006 and first reported by Cano-Sarabia *et al.* [75] in 2008, consists of depressurizing the gas expanded liquid solution into another solvent that acts as a crystallization interruption agent [5]. The authors used this process in the preparation of unilamellar cholesterol vesicles. Briefly, the gas-expanded liquid solution was depressurized from the working pressure to the atmospheric one through a non-return valve over a pumped aqueous solution with 1 wt % surface-active compound. The aqueous solution flow rate was adjusted to fix the cholesterol/surfactant ratio in the final vesicular system. Stable and structurally well-defined uniform spherically shaped, unilamellar rich cholesterol nanovesicles dispersed in an aqueous phase were formed by this process showing physicochemical characteristics unachievable by conventional mixing technologies [75].

All the techniques described in this review are based on the PGSS concept of expanding (or spraying) a gas saturated solution through a restriction device (e.g., a nozzle) and were developed based on one or more limitations with the intention of expanding the applicability of the original patented PGSS technique. Table 1 summarizes the main aspects of different PGSS-related techniques, in which CO_2_ plays different roles and that have been developed since Weidner and co-workers patented the process in 1994.

**Table 1:** Main aspects of different PGSS-based techniques.

Technique	CO_2_ role	Liquid solvents	Pre-requisites	Equilibrium measurements	Saturation	Precipitation	Drying
**PGSS**	Solute	NA	Melted or substances that experiment a mp depression effect under CO_2_ conditions	S-L-G equlibrium	High pressure reactor with mixing or in a static mixer	Spray tower	NA
**CPF**			The solute to be powderized is a liquid	VLE of the binary system (solute + CO_2_)	High pressure reactor with mixing	NA (Infiltration occur in a spray tower)	
**CPCSP**			Melted or substances that experiment a mp depression effect under CO_2_ conditions	S-L-G equlibrium	Static mixer	Spray tower	
**CAN-BD**	Co-solute (aerosolization aid)	Water and alcohol	Limited solubility between scCO_2_ and the liquid solvent	VLE of the binary system (solvent + CO_2_)	CO_2_ solubilisation occur in a low volume tee	Spar tower	With N_2_
**SEA**					CO_2_ solubilisation occur in a pre-expansion mixing chamber	High pressure vessel equipped with a filter	NA
**SAA**					Packed tower	Spay tower	With N_2_
**PGSS-drying**					Static mixer	Spray tower	With CO_2_ (T must be higher than the dew point of the binary system gas and solvent)
**DELOS**	Co-solvent	Organic solvents	CO_2_ acts as a co-solvent	VLE of the ternary system (solute + CO_2_ +organic solvent)	Autoclave	High pressure vessel equipped with a filter	With CO_2_

## 5. Applications of PGSS and PGSS-based Techniques

PGSS and related techniques have been successfully applied to a large range of different substances underlining its enormous versatility. Applications of PGSS and PGSS-based techniques were extensively revised by Perrut in 2001 [14] and by Cor Peters in 2003 [15]. For the CAN-BD and SAA techniques, published applications have further been revised by Sievers in 2008 [48]. The intention of this review is to provide a compilation of all applications published until 2010 in the form of tables (Table 2, Table 3, Table 4 and Table 5) divided by type of process and listing the substances to be micronized in alphabetic order.

**Table 2:** Substances atomized by the PGSS, CPF, CPCSP and PGSS drying techniques.

Substance	Technique	References
Anthocyanin extracts/silica	CPF	Vatai *et al.* (2008) [76]
Caffeine/glyceryl monostearate	PGSS	de Sousa *et al.* (2007) [77]
Caffeine/glyceryl monostearate/cutine/TiO2	PGSS	Garcia-Gonzalez *et al.* (2009) [78]
trans-Chalcone	PGSS	de Sousa *et al.* (2009) [79]
Citrus flavour	CPF	Gruner *et al.* (2003) [80]
Citric acid/PEG	PGSS	Weidner *et al.* (1996) [26]
Cyclosporine	PGSS	Tandya *et al.* (2006) [81]
Coatings systems (acrylic coatings, polyester-epoxy systems, low-melting polyester coatings)	CPCSP	Weidner *et al.* (2001) [43]
Cocoa butter	PGSS	Letourneau *et al.* (2005) [82]
Cocoa powder	PGSS	Perva-Uzunalic *et al.* (2008) [83]
Cilantro(Coriandrum sativum)/PEG		Choi *et al.* (2009) [84]
Cydia pomonella granulovirus	PGSS	Pemsel *et al.* (2010) [85]
Felodipine, Felofipine/lactose, Felodipine/PEG4000	PGSS	Kerc *et al.* (1999) [86]
Fenofibrate, Fenofibrate/PEG4000	PGSS	Kerc *et al.* (1999) [86]
Glutathione/glyceryl monostearate/cutine/TiO2	PGSS	Garcia-Gonzalez *et al.* (2009) [78]
Glyceryl monostearate	PGSS	de Sousa *et al.* (2007) [77]
Green tea extracts (Aqueous)	PGSS-drying	Meterc *et al.*(2008) [64]
hgH/PLGA/PLA	PGSS	Jordan *et al.* (2010) [87]
rh-gH/ Phosphatidylcholine/PEG/Tristearin	PGSS	Salmasso *et al.* (2009) [88]
Hydrogenated palm oil	PGSS	Li *et al.* (2005) [89]
Insulin/tristearin, Tween-80, phosphatidylcholine, PEG, Insulin/tristearin, dioctyl sulfosuccinate and phosphatidylcholine	PGSS	Salmaso *et al.* (2009) [38]
Ketoprofen/glyceryl monostearate/cutine/TiO2	PGSS	Garcia-Gonzalez *et al.* (2009) [78]
Lavandin essential oil/(OSA)-starch	PGSS-drying	Varona *et al.* (2010) [69]
Lavandin essential oil/PEG	PGSS	Varona *et al.* (2010) [69]
Lysozyme/P(DLLA)	PGSS	Whitaker *et al.* (2005) [90]
Monostearate	PGSS	Mandzuka *et al.* (2008) [91]
Nifedipine, Nifedipine/PEG 4000	PGSS	Kerc *et al.* (1999) [86], Sencar-Bozic *et al.* (1997) [28]
Polybutylenterephthalate, Polybutylenterephthalate /zinc oxide, Polybutylenterephthalate/bentonite	PGSS	Pollak *et al.* (2010) [92]
Poly (DL-lactic acid)	PGSS	Hao *et al.* (2004) [37]
Poly (ethylene glycol)	PGSS	Hao *et al.* (2005) [93], Nalawade *et al.* (2007) [94]
Poly (ethylene glycol) aqueous solution	PGSS-drying	Martin *et al.* (2010) [68]
Precirol	PGSS	Calderone *et al.* (2007) [95]
Rapeseed 70	PGSS	Manuklu *et al.* (2007) [96]
PEGylated Ribonuclease/ Triestearin/Phosphatidylcholine/PEG	PGSS	Vezzu et a. (2010) [97]
Ribonuclease A/P(DLLA)	PGSS	Whitaker *et al.* (2005) [90]
Theophylline/hydrogenated palm oil	PGSS	Rodrigues *et al.* (2004) [30]
TiO2-PLA, TiO2-PS-b-PMMA-co-PGMA	PGSS	Matsuyama *et al.* (2007) [98]
Triacetyl-β-cyclodextrin	PGSS	Nunes *et al.* (2010) [99]
Tristearate	PGSS	Mandzuka *et al.* (2008) [91], Mandzuka *et al.* (2010) [100]
Vegetable oil emulsion/cellulose	CPF	Wehowski *et al.* (2008) [101]
YNS3107/PEG400/PEG4000/Polaxamer 407	PGSS	Brion *et al.* (2009) [102]

**Table 3:** Substances atomized with the CAN-BD and SEA processes.

Substance	Liquid solvent	References
Albuterol sulfate	Water	Sievers *et al.* (1998, 2000, 2001) [47,103,104]
Alpha-1-antitrypsin	Water	Cape *et al.* (2008) [48]
Amphotericin B	Ethanol	Sievers *et al.* (2003) [50]
Anti-CD4	Water	Cape *et al.* (2008) [48]
Betamethasone-17,21-dipropionate	Ethanol	Villa *et al.* (2005) [51]
Budesonide	Ethanol	Sievers *et al.* (2003) [50]
Cromolyn sodium	Water	Sievers *et al.* (2000) [47]
Doxycycline	Water	Sievers *et al.* (2003) [105]
Glutathione	Water	Sievers *et al.* (1999) [46]
Myo-inositol	Water	Huang *et al.* (2003) [49]
HBsAg (Hepatitis B surface antigen protein)/Albumin hydroxide	Water	Sievers *et al.* (2007) [52]
Iron oxides mixture (Fe3O4 and FeO)	Water	Sievers *et al.* (1999) [46]
Lactate dehydrogenase (LDH)	Water	Sellers *et al.* (2001) [106], Sievers *et al.* (2001) [104]
Lactose	Water	Sievers *et al.* (2000) [47], Villa *et al.* (2005) [51]
Lactose/Betamethasone	Water/Ethanol	Villa *et al.* (2005) [51]
Lactose/( Betamethasone/Stearic acid)	Water/Ethanol	Villa *et al.* (2005) [51]
Lactose/Palmitic acid	Water/Ethanol	Villa *et al.* (2005) [51]
Lysozyme	Water	Sellers *et al.* (2001) [106], Sievers *et al.* (2001) [104]
Mannitol	Water	Huang *et al.* (2003) [49]
Measles Vaccine virus, live-attenuated	Water	Sievers *et al.* (2007) [52], Burger *et al.* (2008) [53]
Naproxen	Water	Sievers *et al.* (2003) [50]
Ovalbumin/trehalose	Water	Sievers *et al.* (2001) [104], Sievers *et al.* (2003) [50]
Palmitic acid	Ethanol	Villa *et al.* (2005) [51]
rhDNase	water	Sievers *et al.* (1999) [46]
Rifampin	Ethyl acetate	Sievers *et al.* (2007) [52]
Sacharin(SAC)-Aspirin, SAC-Caffeine, SAC-Carbamazeoine, SAC-Indomethacin, SAC-Sulfamethazine, SAC-Theophylline (Cocrystals)	Ethanol	Padrela *et al.* (2009) [61], Padrela *et al.* (2010) [62]
Sodium chloride	Water	Sievers *et al.* (2001) [104], Sievers *et al.* (2003) [50], Villa *et al.* (2005) [51]
Sodium chloride/Palmitic acid	Water/acetone	Villa *et al.* (2005) [51]
Sodium chloride/PLGA	Water/acetone	Villa *et al.* (2005) [51]
Tobramycin sulfate	Water	Sievers *et al.* (1998) [103]
Trypsinogen	Water	Cape *et al.* (2008) [48]
Yttrium oxide phosphors (Y2O3:Eu, Y2O3:Tb)	Water	Xu *et al.* (1997) [45]
Zanamivir (Relenza^®^)	Water	Sievers *et al.* (2007) [52]

**Table 4:** Substances atomized with the SAA process.

Substance	Liquid solvent	References
Albumin/Gentamicin sulfate	Water	Della Porta *et al.* (2010) [107]
Aluminum sulfate	Water	Reverchon *et al.* (2002) [54]
Amonium chloride	Water	Reverchon *et al.* (2004) [108]
Ampicillin	Water, methanol, ethanol	Reverchon *et al.* (2002, 2003) [54,109]
Ampicillin trihydrate /Chitosan	Water	Reverchon *et al.* (2007) [110]
HPMC/ampicillin trihydrate	Buffer solution	Reverchon *et al.* (2008) [111]
Beclomethasone	Methanol, acetone, methanol/water, acetone/water	Reverchon *et al.* (2010) [112]
Carbamazepine	Methanol	Reverchon *et al.* (2002) [54]
Cefadroxil	Water	Li *et al.* (2009) [113]
Chitosan	1%acid acetic aqueous solution	Reverchon *et al.* (2006) [114]
Cromolyn Sodium	Water	Reverchon *et al.* (2007) [115]
α-Cyclodextrin	Water	Reverchon *et al.* (2006) [116]
Dexamethasone, Dexamethasone acetate	Acetone, methanol	Reverchon *et al.* (2002, 2006) [54,117]
Erythromycin	Methanol, ethanol, acetone	Reverchon *et al.* (2003, 2004) [118,119], Li *et al.* (2007) [120]
Ginkgo biloba leaves extract	x	Miao *et al.* (2010) [121]
Griseofulvin	Acetone, acetone/ethanol	Reverchon *et al.* (2004) [122], Li *et al.* (2008) [123]
HP-beta-CD	Water	Reverchon *et al.* (2006) [114]
HMR1031 (new chemical entity by Aventis Pharma)	Methanol	Reverchon *et al.* (2005) [124]
Levofloxacin hydrochloride	Methanol	Cai *et al.* (2008) [60]
Lysozyme	Water, water/ethanol mixtures	Reverchon *et al.* 2009 [125]
Pigment red 60	Acetone	Reverchon *et al.* (2005) [126]
PLLA	DCM	Reverchon *et al.* (2007) [127]
PMMA	Acetone	Reverchon *et al.* (2007) [127]
Potassium iodide	Water, methanol	Reverchon *et al.* (2004) [108]
Rifampicine	Methanol	Reverchon *et al.* (2003) [128]
Sodium chloride	Water	Reverchon *et al.* (2002, 2004) [54,108]
Sodium cellulose sulfate	Water	Wang *et al.* [129]
Terbutaline	Water	Reverchon *et al.* (2003) [130]
Tetracycline	Water, water/ethanol	Reverchon *et al.* (2003) [128,119], Li *et al.* 2008 [131]
Triclabenzadol	Methanol	Reverchon *et al.* (2002) [54]
Yttrium acetate	Water, methanol	Reverchon *et al.* (2002, 2003) [54,119]
Zinc acetate	Methanol	Reverchon *et al.* (2002) [54]
Zirconyl nitrate hydrate	Water	Reverchon *et al.* (2002) [54]

**Table 5:** Substances atomized with the DELOS process.

Substance	Liquid solvent	References
1,4-bis-(n-butylamino)-9,10-anthraquinone (solventblue35)	Acetone	Ventosa *et al.* (2001, 2003) [71,72]
1,4-bis-(n-butylamino)-9,10-anthraquinone (solventblue35)	1,1,1,2-Tetrafluoroethane	Gimeno *et al.* (2006) [132]
1,3,5,7-Tetraazatricyclo[3.3.1.13,7]decane (hexamethylenetetramine)	1,1,1,2-Tetrafluoroethane	Gimeno *et al.* (2006) [132]
Acetylsalicylic acid (aspirin)	1,1,1,2-Tetrafluoroethane	Gimeno *et al.* (2006) [132]
Cholesterol		Cano-Sarabia *et al.* (2008) [75]
Ibuprofen	Ethanol and Acetone	Munto *et al.* (2008) [133]
Naproxen	ethanol	Munto *et al.* (2008) [133]
Poloxamer F-127	ethanol	Munto *et al.* (2008) [134]
Stearic acid	Ethyl acetate	Sala *et al.* (2010) [135]

## 6. Conclusions and Future Perspectives

Since Weidner and co-workers patented the PGSS process in 1994, several variations based on the same concept were developed in which dense CO_2_ plays different roles; as a solute in CPF and CPCSP; as a co-solute for CAN-BD, SEA, SAA and PGSS-drying; and as a co-solvent in the DELOS process. PGSS-derived techniques, besides offering several advantages over conventional processes, are based on the very simple concept of expanding (or spraying) a solution saturated with a dense gas through a restriction device (e.g., a nozzle). The concept has actually proven its feasibility as PGSS is already operating on large scales for producing products for the food industry. Nevertheless, most published papers presented in this review explore applications directed to the pharmaceutical industry, which is in general more conservative when it comes to technological changes. For example, the widely in use spray drying process had only started to be employed by the pharmaceutical industry twenty years after it found its first industrial application in the food industry, for milk drying [136]. It is therefore very likely that, in coming years, PGSS-based techniques will find their way into the pharmaceutical industry. The research road ahead is as important as the one done until this point, but is essentially more demanding. A huge number of publications have evidenced the versatility of these techniques in allowing the processing of several different types of substances and, although the development of new products remains important, it is crucial to understand some process mechanisms that are still not fully understood. Even though some efforts have been done in the past few years, some fundamental issues still require further research in order to better understand the process mechanisms involved. The development of models that can accurately predict the characteristics of the final product constitute the great challenge that scientists in the field have to address, so that the technology can become widespread.

## Supplementary Files

Supplementary File 1
