# Correction: Dense CO_2_ as a Solute, Co-solute or Co-solvent in Particle Formation Processes: A Review. *Materials* 2011, *4*, 2017-2041.

**DOI:** 10.3390/ma5061114

**Published:** 2012-06-18

**Authors:** Ana V.M. Nunes, Catarina M.M. Duarte

**Affiliations:** 1 Requimte/CQFB, Departamento de Química, Faculdade de Ciências e Tecnologia Universidade Nova de Lisboa, Campus de Caparica, Caparica 2829-516, Portugal; E-Mail: ana.nunes@dq.fct.unl.pt; 2 Instituto de Biologia Experimental e Tecnológica (IBET), Apartado 12, Oeiras 2781-901, Portugal; 3 Instituto de Tecnologia Química e Biológica, Universidade Nova de Lisboa, Avenida da Republica, Oeiras 2780-157, Portugal

Due to a lapse the Acknowledgements section was missed from the original article version.

