# Supplementary material for: Dense CO2 as a Solute, Co-Solute or Co-Solvent in Particle Formation Processes: A Review
Source: Materials (Basel). 2011 Nov 16;4(11):2017–41. doi: 10.3390/ma4112017 (PMC5448852; doi:10.3390/ma4112017)
Supplement: Supplementary File 1 [file materials-04-02017-s001.pdf]

Materials **2012**, *5*, 1114; doi:10.3390/ma5061114

OPEN ACCESS

materials

ISSN 1996-1944

www.mdpi.com/journal/materials

Correction

Correction: Dense CO_2 as a Solute, Co-solute or Co-solvent in Particle Formation Processes: A Review. *Materials* 2011, *4*, 2017-2041.

Ana V.M. Nunes [1] and Catarina M.M. Duarte [2,3,]*

[1] Requimte/CQFB, Departamento de Química, Faculdade de Ciências e Tecnologia Universidade Nova de Lisboa, Campus de Caparica, Caparica 2829-516, Portugal;
E-Mail: ana.nunes@dq.fct.unl.pt

[2] Instituto de Biologia Experimental e Tecnológica (IBET), Apartado 12, Oeiras 2781-901, Portugal

[3] Instituto de Tecnologia Química e Biológica, Universidade Nova de Lisboa, Avenida da Republica, Oeiras 2780-157, Portugal

* Author to whom correspondence should be addressed; E-Mail: cduarte@itqb.unl.pt;
Tel.:+351-214-469-727; Fax: +351-214-421-161.

Received: 29 May 2012 / Accepted: 18 June 2012 / Published: 18 June 2012

Due to a lapse the Acknowledgements section was missed from the original article version.

Acknowledgments

The authors would like to thank to Fundação para a Ciência e Tecnologia, Portugal, for financing the Project PTDC/EQU-EQU/104552/2008. Ana Nunes further acknowledges its post-doc grant SFRH / BPD / 74994/ 2010.

© 2012 by the authors; licensee MDPI, Basel, Switzerland. This article is an open access article distributed under the terms and conditions of the Creative Commons Attribution license (http://creativecommons.org/licenses/by/3.0/).
